# Maternal anxiety shapes prediction error responses in the infant brain

**DOI:** 10.1117/1.NPh.12.3.035013

**Published:** 2025-09-16

**Authors:** Addison D. N. Billing, Eleanor S. Smith, Robert J. Cooper, Rebecca P. Lawson

**Affiliations:** a University of Cambridge, Department of Psychology, Cambridge, United Kingdom; b DOT-HUB, University College London, Department of Medical Physics and Biomedical Engineering, London, United Kingdom

**Keywords:** fNIRS, gaze-tracking, high-density diffuse optical tomography, learning, probabilistic association learning, prediction

## Abstract

**Significance:**

Postnatal maternal anxiety affects a substantial number of new mothers and is linked to long-term risk for anxiety in their offspring. Yet, the neural mechanisms through which postnatal maternal anxiety influences early cognitive development remain unclear. We investigated whether postnatal maternal anxiety shapes how infant brains respond to unexpected events—prediction errors—which are central to learning in uncertain environments.

**Aim:**

We examined prediction error processing in 6- to 8-month-old infants using high-density diffuse optical tomography and eye-tracking. We hypothesized that neural responses in the medial prefrontal cortex (mPFC) would vary with maternal anxiety levels.

**Approach:**

Infants viewed audiovisual events where expected outcomes were occasionally omitted, eliciting prediction errors. Hemodynamic responses in the frontal cortex were analyzed using a general linear model, with trial-by-trial gaze data as a parametric modulator. Maternal anxiety was measured using the state-trait anxiety inventory.

**Results:**

Prediction error responses were localized to the mPFC and were only detectable when controlling for infant attention using eye-tracking. Cortical activation in response to unexpected stimuli was significantly enhanced in infants of mothers with higher trait anxiety.

**Conclusion:**

Our findings suggest that maternal anxiety modulates prediction error processing in the infant brain, potentially shaping early sensitivity to environmental unpredictability and conferring risk for later anxiety.

## 1 Introduction

Post-natal maternal anxiety affects a substantial proportion of new mothers, with prevalence rates reaching up to 34.2%.^1^ Despite this, in the WHO European region, only 11 out of 53 countries offer any type of perinatal mental health treatment service, and only 10 countries have established perinatal mental health screening.^2^ Postnatal maternal anxiety is associated with an increased risk of anxiety and emotional dysregulation in offspring,^3^^,^^4^ yet the mechanisms underpinning this transmission remain incompletely understood.^5^ Although anxiety disorders have a moderate heritability (∼30% to 50%), twin studies suggested that genetic factors alone do not fully account for intergenerational transmission.^3^ Instead, genetic vulnerability may interact with early environmental influences to shape the development of anxiety-related traits. For example, children with a high polygenic risk for anxiety may be particularly sensitive to maternal emotional states, amplifying the effects of exposure to an unpredictable or threat-sensitive caregiving environment.^6^

The early environment, particularly the consistency and predictability of parental cues, plays a crucial role in shaping infant cognitive and affective development. According to predictive processing models of perception, the brain continuously generates expectations about incoming sensory input and updates these expectations based on experience.^7^^,^^8^ Unexpected events—those that violate prior expectations—produce prediction errors, which signal the need for learning or adaptation. However, in environments characterized by unpredictability, infants may learn that their surroundings are unstable, leading to increased sensitivity to unexpected stimuli. This aligns with findings in adult anxiety, where heightened neural responsivity to prediction errors has been proposed to underlie increased threat anticipation and intolerance of uncertainty.^9^^,^^10^ Despite growing evidence that maternal anxiety influences infant socio-emotional development,^4^^,^^11^ little is known about its effects on early neural mechanisms of surprise processing.

Probabilistic associative learning is the process by which individuals learn to associate specific cues with the likelihood of particular outcomes based on probabilistic information.^12^ This learning relies on prediction errors—the discrepancy between expected and actual outcomes—that serve as signals to update future predictions. Although prediction error signals can be measured in various cortical regions depending on the task (e.g., perceptual areas for perceptual prediction errors), large-scale meta-analyses show that their domain-general processing occurs in fronto-subcortical circuits, which support learning.^13^ Convergent evidence across rodents,^14^ nonhuman primates,^15^ and human adults^16^^–^^18^ indicates that the medial prefrontal cortex (mPFC), in particular, plays a key role in error detection. Computational neuroimaging studies suggest that the mPFC is particularly sensitive to volatility—the degree to which an environment is unpredictable—dynamically encoding expectation violations and adjusting learning rates in response to changing contingencies.^19^^,^^20^ Within this framework, anxiety has been linked to dysregulated mPFC activity during prediction error processing,^21^ which can interfere with learning and decision-making in uncertain contexts.

Importantly, developmental research suggested that early-life experiences with anxiety may shape the maturation of mPFC circuits involved in learning and expectation updating.^22^ It is therefore possible that infants raised in high-anxiety environments may develop heightened neural responsivity to unexpected stimuli as their mPFC adapts to an unstable caregiving context by prioritizing threat-related prediction errors. Given that infant learning is highly dependent on probabilistic environmental cues,^23^ altered prediction error processing in the context of maternal anxiety may have long-term consequences for cognitive flexibility, emotional regulation, and risk for anxiety disorders.

However, methodological limitations make it challenging to measure neural prediction error responses in awake, behaving infants. Pioneering studies have used low-resolution functional near-infrared spectroscopy^24^^,^^25^ to study infants from 5 months old, showing evidence of prediction error such as responses in sensory cortices—activation for unexpected stimulus omissions equivalent to stimulus presence. However, no studies to date have examined the function of the mPFC in infants within the context of prediction error processing, potentially owing to limits on spatial resolution, field of view, and signal-to-noise ratio in conventional near-infrared spectroscopy (fNIRS) systems.

High-density diffuse optical tomography (HD-DOT) is an advanced optical imaging method that uses dense, spatially overlapping measurements to generate 3D reconstructions of brain activity. Its dense optode array improves cortical mapping, distinguishes adjacent functional regions, and extends near-infrared light penetration for deeper activity detection, with a spatial resolution approaching that of fMRI.^26^^,^^27^ HD-DOT is well-tolerated in infants^28^ and, with its high temporal resolution, is ideal for studying trial-by-trial fluctuations in cognitive processes such as learning. Infant fNIRS data are often noisy, leading researchers to average trials by event type (e.g., expected versus unexpected), which disregards dynamic processes such as repetition suppression,^29^ belief updating, and prediction error-driven attention shifts.^30^ Attention is a particularly important variable in infant neuroimaging as “unexpected” trials have typically involved stimulus omissions,^24^ and when nothing is on the screen, infants are more likely to look away toward something more salient. Similarly, stimulus repetition, as in the “expected” trials, reduces novelty, which is also likely to drive reductions in attention. These moment-to-moment fluctuations are best captured with concurrent eye-tracking as an objective measure of task engagement,^31^ which can be factored into imaging analyses.

The primary aim of this study was to examine whether prediction error processing in the infant frontal cortex varies as a function of maternal anxiety, potentially providing insight into early neurocognitive mechanisms underlying anxiety transmission. Here, we examine prediction error processing in infants from 6 to 8 months old, capitalizing on an event-related design approach to address trial-level variability in hemodynamics. We use concurrent eye tracking to measure infant engagement with the task across trials, included as a parametric modulator of hemodynamic responses in the context of a generalized linear modelling (GLM) analysis framework. We designed an optode array optimized for dense frontal cortex sampling, with long-range connections across hemispheres to capture medial sources. Maternal anxiety was measured via the state-trait anxiety inventory (STAI^32^). Our aim was to examine frontal processing of prediction errors at a level of detail never before achieved in infants. We hypothesized that neural responses in the mPFC would show greater activation to unexpected compared with expected events and that the magnitude of this prediction error response would be positively associated with maternal trait anxiety levels.

## 2 Methods

### 2.1 Participants

Forty-one babies were tested using concurrent eye-tracking and HD-DOT. Of these infants, six were removed from the dataset due to poor-quality eye-tracking data. The experimenter forgot to place the optical shielding cover on the head of one infant, thus excluding that infant from the dataset. Three infants did not meet the requirement of having a minimum of three of each trial type and were also removed. The final sample therefore included 31 babies between 6 and 8 months (M age = 215 days; SD=16 days, 19 female).

Sample size was determined by practical constraints of infant neuroimaging research. Based on effect sizes reported in similar infant prediction error paradigms (Emberson et al.^24^; $\eta^{2}=0.31$ for main effects), our sample size (n=31 for main analyses, n=25 for maternal anxiety analyses) provides ∼85% power to detect large effects (f=0.35) and 65% power for medium-large effects (f=0.25) using repeated measures ANOVA with α=0.05. Although modest, this falls within the typical range for infant fNIRS studies and represents adequate power for the effect sizes typically observed in developmental neuroimaging research.

The experiment was performed at Cambridge BabyLab within the Craik Marshall building at the Cambridge University Department of Psychology. Recruitment was carried out using the Cambridge Babylab Infant Database as well as through social media (Facebook and Twitter). Parents were paid for travel expenses, and written, informed parental consent was obtained for each participant prior to their participation (Cambridge Psychology Research Ethics Committee approval: PRE.2019.106).

### 2.2 Stimuli and Experimental Design

Following prior research,^24^ we adapted the paradigm for our event-related design and frontal cortex focus. We deliberately modified the paradigm from Emberson et al.^24^ to optimize it for our specific research goals: (1) our event-related design with concurrent eye-tracking required precise temporal control and shorter trial durations; (2) our primary interest was in frontal prediction error responses rather than sensory cortical responses; (3) for frontal regions, prediction error signals are more likely driven by expectation violation rather than simple visual stimulation differences.

**Fig. 1:**
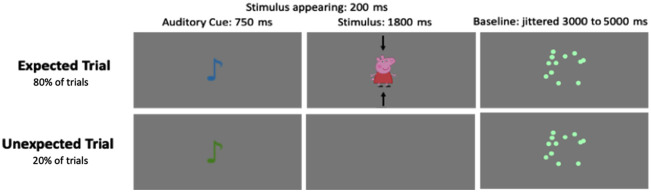
Experimental paradigm for infant prediction error study. The test paradigm is designed to elicit prediction error responses in infants by violating expectations established during the familiarization phase. A 750 ms sound clip is played, followed by a 1800 ms cartoon pig appearance (80% of the time) or no pig appearance (20% of the time). There is a jittered baseline of a 3 to 5 s video of bubbles popping between trials.

Infants first underwent a brief familiarization phase to associate hearing a sound clip (750 ms) with a cartoon pig appearing on the screen (1800 ms). The audio cue and associated cartoon were played 10 times, and the cartoon pig could arrive from either the top or the bottom of the screen (counterbalanced). In the experimental phase, the learned audio cue was presented, followed by the cartoon pig appearing 80% of the time (“expected trials”). On the 20% occasions that the pig did not appear (“unexpected trials”), this was cued by a different audio clip. In the experimental phase, there was a jittered baseline video of bubbles popping, lasting between 3 and 5 s (Fig. 1). The unequal trial numbers (expected: M=22.65, unexpected: M=4.81) reflect the probabilistic nature of prediction error paradigms, where unexpected events must be relatively rare to maintain their “unexpected” quality and prevent habituation. On average, the infants saw the expected trials 22.65 times (mode = 22, median = 21, range = 16 to 36). Unexpected trials were viewed an average of 4.81 times (mode = 4, median = 4, range = 3 to 8).

During the experiment, infants sat on their caregiver’s lap, or in a highchair in a dimly lit room approximately 60 cm distance from the screen. Parents were asked to limit communicating with their infant during the paradigm. The testing session ended if the infants became fussy. A five-point eye-tracking calibration was used, which included a series of calibration characters (i.e., butterfly, bumblebee) set on a brightly colored circular background (presented at 4 cm and shrinking to 1 cm), which were presented alongside an interactive noise (e.g., “boing”) to attract the infant’s attention.

### 2.3 Data Acquisition

Visual stimuli were presented on a screen with 1920×1080 resolution using OpenSesame version 3.2.8. Eye-tracking data were captured by an EyeLink Portable Duo eye-tracker (SR Research) sampling at 100 Hz, recorded using OpenSesame—Pygaze/PyLink integration version 0.6.0a26, and analyzed using EyeLink DataViewer and custom MATLAB scripts.

**Fig. 2:**
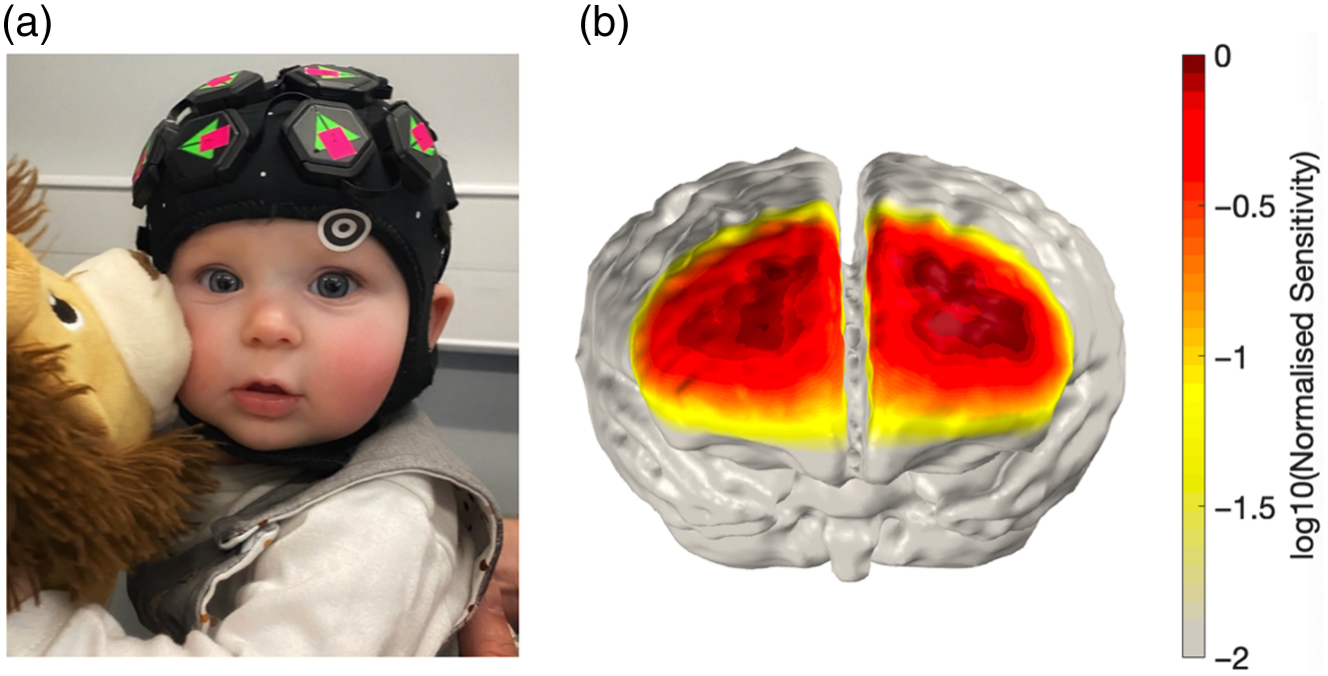
Cap sensitivity. The image depicts the placement (a) and sensitivity (b) of the frontal tiles from the LUMO (HD-DOT) cap used during the experiment. This configuration maximizes sensitivity to the medial prefrontal cortex.

HD-DOT data were collected at 4.6 Hz using a LUMO device (Gowerlabs Ltd., UK). The 12-tile LUMO cap was designed to be maximally sensitive to the frontal lobes, with some coverage of primary occipital and parietal regions, providing 1728 logical channels per wavelength, of which 596 were expected to be within the measurable range of source-detector separations (0 to ∼50  mm). This configuration maximizes sensitivity to the medial prefrontal cortex (Fig. 2). For each infant, the correct placement of the cap in relation to anatomical landmarks was verified before and after the paradigm. A custom light-shielding material was placed over the LUMO cap to minimize any interference from the optical sources of the eye-tracker. Head circumference, auricular-to-auricular (al-ar), and nasion-to-inion (nav-iz) measurements were taken to accurately place the head cap. Head circumference measurements were collected across all infants (M=44.5  cm, SD=1.41).

After the session, parents filled out a questionnaire online, which included the state-trait Anxiety inventory (STAI).^32^ The STAI has well-established validity and is one of the most widely used measures of anxiety symptoms.^33^ The STAI has been used in perinatal samples with clinical cut-offs of >40.^34^

Optode positions were determined using a standardized cap placement protocol based on the international 10 to 20 electrode placement system landmarks (nasion, inion, left and right preauricular points, Cz, Fpz), which were individually measured for each participant. Head circumference, auricular-to-auricular (al-ar), and nasion-to-inion (nav-iz) measurements were taken to accurately place the head cap. We used the Colin-27 adult atlas for ROI definition. Although an infant-specific atlas would be ideal, this was not available within our analysis toolbox at the time. However, we do not expect this to significantly impact our results because: (1) the frontal regions of interest show relatively mature sulcal patterns by 6 to 8 months; (2) our analysis used a weighted sensitivity approach that accounts for individual head size variations based on the 10 to 20 landmark measurements; (3) individual anatomical variability within our infant sample and practical measurement uncertainties from cap placement likely exceed any systematic differences between adult and 6 to 8 month brain templates; and (4) the spatial resolution of HD-DOT and our broad frontal ROI definitions are not sufficiently precise to be substantially affected by subtle template differences. Although some uncertainty in precise anatomical localization remains when using adult templates for infant data, the use of individualized 10 to 20 landmark measurements and comprehensive head measurements (circumference, al-ar, nav-iz) helps minimize systematic errors. Given the individual anatomical variability within our sample and the spatial resolution of our methods, we do not expect template choice to substantially affect our conclusions about frontal cortical responses.

### 2.4 Analysis

Dwell time ratio (DTR) was calculated as the proportion of time infants maintained gaze within a screen-centered area of interest (AOI) spanning the full display area (1920×1080  pixels) between auditory onset and the end of the expected stimulus presentation window. Periods of signal loss were handled by calculating DTR as valid gaze time within the AOI divided by total valid tracking time during the trial window, following recommendations by Wass et al.^35^ DTR values were used as a parametric modulator in the analysis rather than as an exclusion criterion.

DOT images were created following a pipeline previously shown to be effective for infant LUMO data.^28^ Channels were rejected if they had a source-detector separation greater than 60 mm or if their coefficient of variation exceeded 8% during time periods with no motion artifacts.^36^ The Homer function hmrMotionArtifactByChannel was used to identify motion artifacts, with the parameters tMotion = 1, tMask = 1, STDEV = 15, AMPthresh = 0.4.^37^^,^^38^ Motion artifacts in the data were corrected using hmrMotionCorrectSpline, (p=0.99) and hmrMotionCorrectWavelet (IQR = 0.8), for optimal recovery of the infant HRF.^37^^,^^39^^,^^40^ To remove slow drifts and cardiac noise, the data were band-pass filtered in the range 0.01 to 0.5 Hz using a FIR digital filter. Channel-wise changes in hemoglobin concentration were obtained using the modified Beer–Lambert–Bouguer law, with a differential pathlength factor (DPF = 5.1) appropriate for both wavelengths in the infant population.^41^^,^^42^ The relative changes in oxyhemoglobin (HbO) and deoxygenated hemoglobin (HbR) were computed for 9-s long epochs starting from 2 s before the onset for expected and unexpected conditions. The average HbO and HbR values during the 2 s pre-onset were used as the baseline against which changes were determined. Group-level responses were obtained by averaging all the time-course blocks across all subjects. In the image analysis, the group-level responses did not include any individual eye-tracking information.

Image reconstruction followed a pipeline previously used with HD-DOT infant data.^28^ Images were reconstructed using a four-layer mesh model of the 6-month infant head. TOAST++ was used to model optical fluence and calculate a Jacobian matrix for each wavelength.^43^ A zeroth-order Tikhonov regularized reconstruction was performed using a regularization hyperparameter of 0.01 for the full mesh volume.

Six regions of interest were analyzed: left and right medial prefrontal cortex (mPFC), left and right medial superior frontal gyrus (mSFG), and left and right middle prefrontal cortex, defined using the Colin-27 atlas and weighted average method described by Zhai et al.^44^ We used 3D coordinates on a phantom with a head circumference of 45 cm to determine where each channel sampled on the cortex. Using these 3D coordinates, it was possible to convert 832 channels (416 for each chromophore between 12 and 45 mm) into regions of interest in the weighted average method described by Ref. 44. Briefly, the optical forward model is used to determine the sensitivity of each channel to a given region on the Colin-27 atlas using the boundary element method.^45^

All timeseries data were trimmed to include only 2 s before the first event and 3 s after the final event ended. Data were resampled to 4 Hz, converted to changes of absorbance (optical density), and motion artifacts were corrected using temporal derivative distribution repair (TDDR).^46^ Changes in hemoglobin concentration determined using the modified Beer–Lambert–Bouguer law (DPF = 5.1) were compiled into a single timeseries per ROI in the method described above.^44^

To test hemodynamic responses to expected and unexpected events, we used a linear mixed-effects model where neural responses (beta values) were predicted by condition (expected versus unexpected), with random intercepts for each subject. We used finite impulse response (FIR) deconvolution and autoregressive pre-whitening iteratively reweighted least squares (AR(P)IRLS), a method that pre-whitens the data to remove temporal correlations and then uses iterative reweighting to reduce the influence of outliers in time series data.^47^ The FIR model had 28 “bins,” which means that the full response was divided into 28 time-points, resulting in 28 beta values. Beta values were averaged between the 12th and 24th bins (3 to 6 s post-stimulus) as this corresponds to the typical peak of the infant hemodynamic response function based on prior research.^28^ The results were corrected for multiple comparisons using the Benjamini–Hochberg method^48^ with a false discovery rate of 0.05. The correction factor was determined based on the total number of statistical tests performed across all ROIs and contrasts (6 ROIs × 2 contrasts = 12 comparisons for the main analysis).

In addition to the condition regressors, a single trial-by-trial parametric modulator was included to account for infant engagement. This regressor was derived from the DTR, defined as the proportion of time the infant maintained gaze during the stimulus presentation window (from auditory onset through the expected visual stimulus duration). DTR values were scaled between 0.1 and 1 prior to convolution with the canonical infant hemodynamic response function. This modulator was applied across all trials, irrespective of condition, allowing the model to account for variability in neural responses due to fluctuations in attention and motivation without assuming condition-specific engagement effects.

For the maternal anxiety analysis, we used a linear mixed-effects model where neural response (beta) was predicted by condition (expected versus unexpected), the interaction between condition and trait anxiety, with random intercepts for each subject to account for individual differences. Infants were only included in this specific analysis relating to maternal anxiety if their mothers, rather than their fathers, completed the assessment (n=25). We used a canonical HRF approach (4-s time-to-peak) for this analysis to increase statistical power by reducing the number of parameters estimated, given our smaller sample size for this specific analysis. To test if an infant’s response to unexpected stimuli in the right mPFC could predict the likelihood of their mothers having clinically diagnosable anxiety levels, we used a logistic regression model with the STAI clinically diagnosable anxiety levels at 40.^34^ The effects of state anxiety in the right mPFC were examined using a similar model structure.

## 3 Results

### 3.1 Eyetracking

**Fig. 3:**
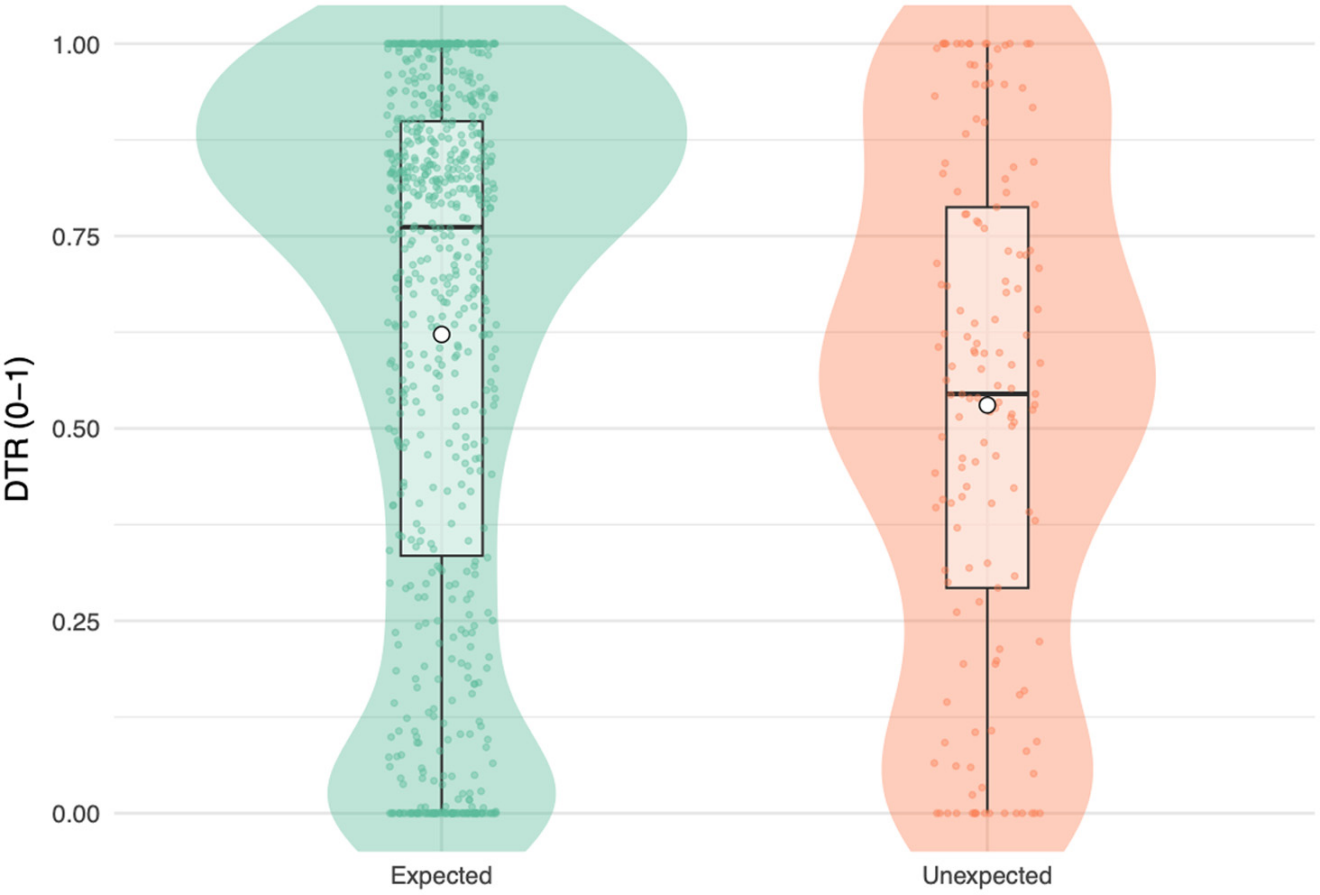
Dwell time ratio by condition. Violin and box plots display the distribution of DTRs for expected and unexpected trials. The plots illustrate that infants exhibit higher DTRs on expected trials compared with unexpected trials. Mean values are indicated by white-filled points.

To test whether engagement differed between conditions, we fitted a linear mixed-effects model with condition (expected vs. unexpected) as a fixed effect and a random intercept for subject to explain the DTR data (Fig. 3). There was a significant main effect of condition, b=−0.10, SE=0.023, t(819.14)=−4.39, p<0.001, such that infants spent significantly less time looking at the screen during unexpected trials (when the cartoon pig did not appear).

To assess the explanatory power of the model, we calculated marginal and conditional $R^{2}$ values using the r.squaredGLMM() function from the MuMIn package. The marginal $R^{2}$ ($R^{2}$ marginal = 0.012) reflects the proportion of variance in DTR explained by the fixed effect of condition alone, indicating that condition accounts for only a small amount of variability across trials. However, the conditional $R^{2}$ ($R^{2}$ conditional = 0.48) reflects the variance explained by both fixed and random effects, demonstrating that between-subject differences contributed substantially to the overall model fit.

**Fig. 4:**
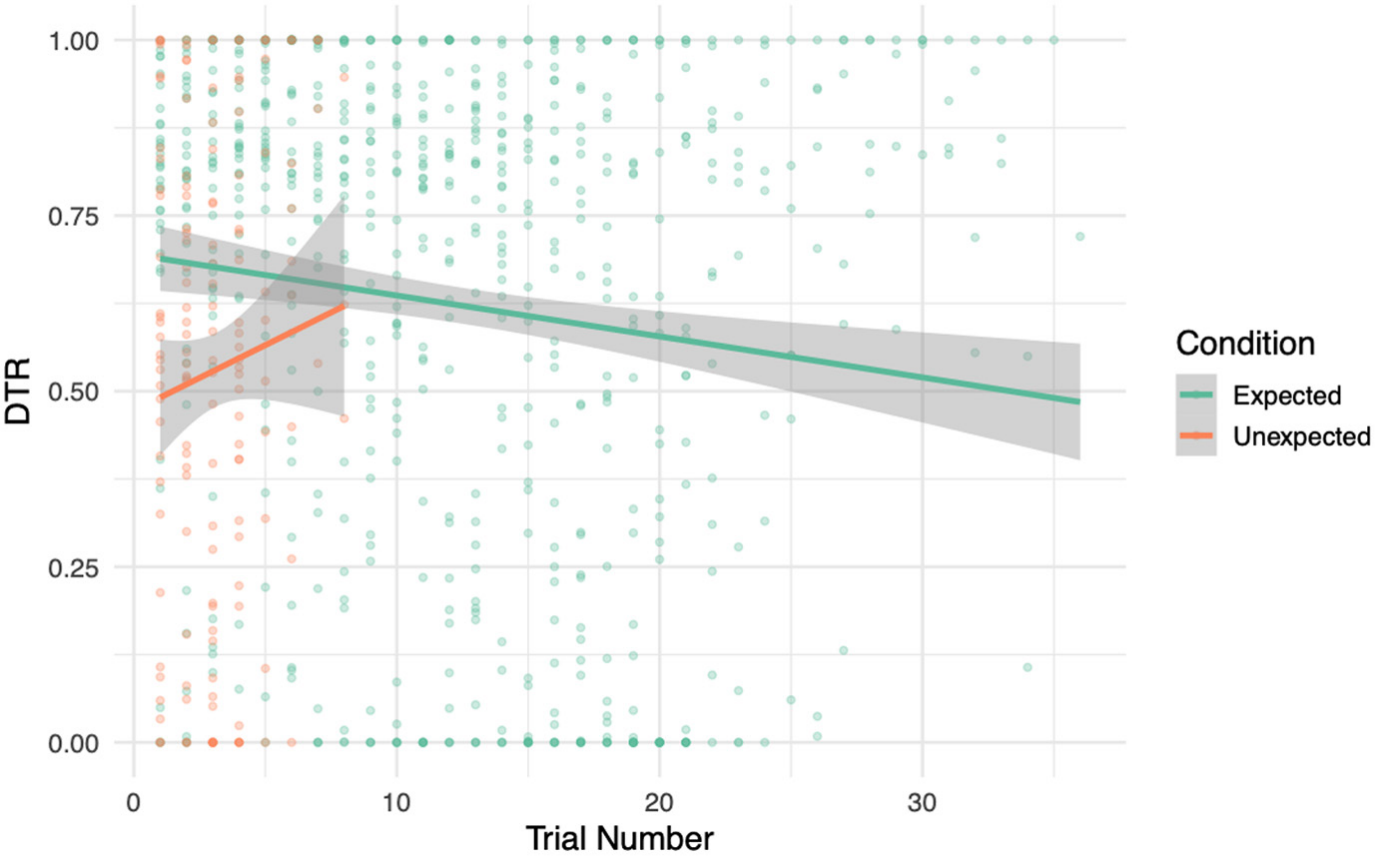
DTR over trial number. A scatter plot with overlaid regression lines shows the relationship between trial number and DTR, separately for expected and unexpected conditions.

We next assessed whether DTR changed as a function of trial number to examine habituation (Fig. 4). A second linear mixed-effects model was fitted with trial number, condition, and their interaction as fixed effects. There was a significant main effect of trial number, b=−0.014, SE=0.001, t(822.24)=−11.43, p<0.001, indicating that gaze duration declined over the course of the experiment—consistent with habituation. The main effect of condition remained significant, b=−0.20, SE=0.043, t(817.04)=−4.69, p<0.001. Although visual inspection of the trial-wise trajectories suggested that DTRs may have declined steeply for expected trials and remained relatively stable, or even slightly increased, for unexpected trials, the interaction between trial number and condition was not statistically significant (b=−0.010, SE=0.011, p=0.37). This indicates that the apparent divergence in habituation slopes across conditions was not reliable at the group level. The habituation model explained more variance overall, with $R^{2}$ marginal = 0.085 and $R^{2}$ conditional = 0.59. This suggests that trial number and condition together accounted for a modest portion of DTR variance, while subject-level variability remained a major contributor. Collectively, these findings underscore the value of including DTR as a parametric modulator because they demonstrate that assuming uniform cortical responses across individuals within each condition would overlook substantial variability in attention and engagement. Shapiro–Wilk tests on model residuals were significant (condition model: W=0.987, p<0.001; habituation model: W=0.993, p<0.001), but Q−Q plots and histograms (Figs. S3 and S4 in the Supplementary Material) showed only minor deviations from normality, supporting the use of linear mixed-effects modelling.

### 3.2 Preliminary Image Space Analysis

**Fig. 5:**
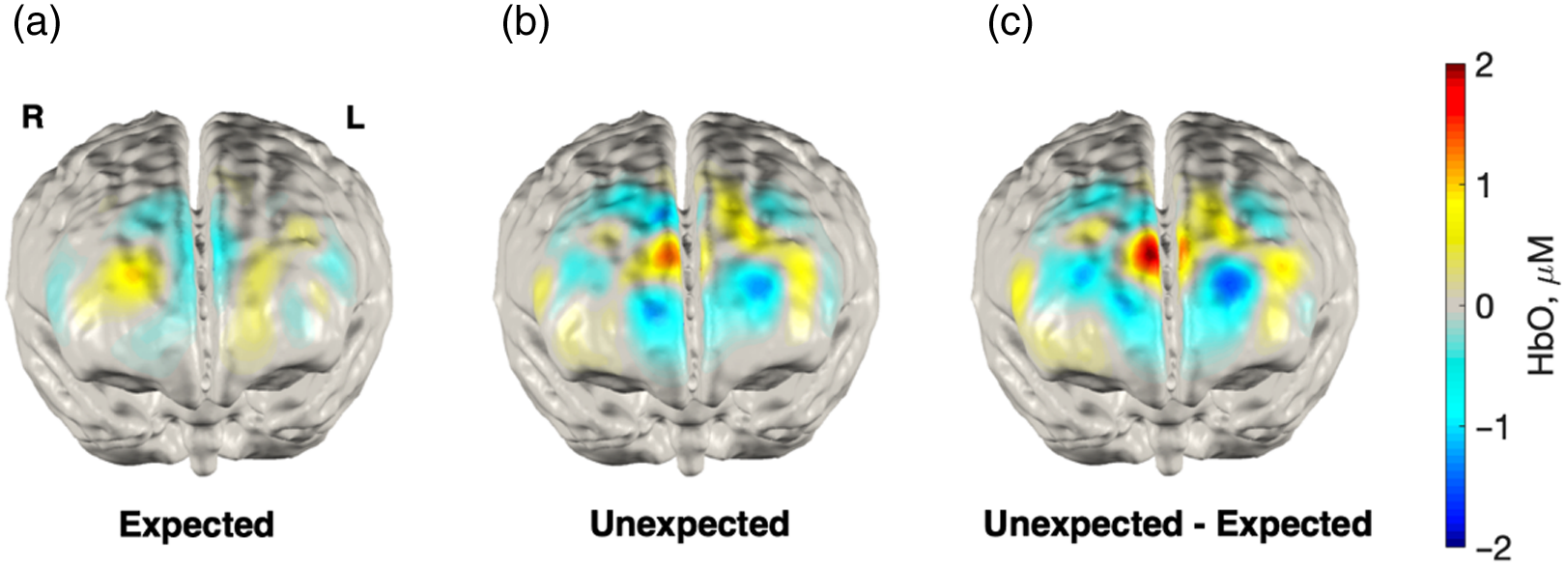
Hemoglobin concentration maps for the frontal lobe. Oxygenated hemoglobin maps are shown for the average expected event contrasted versus baseline (a) the average unexpected event contrasted versus baseline (b) and the contrast of the events (c) over a 3 to 6 s window.

We hypothesized that cortical prediction error responses would localize to the frontal lobe, with specific predictions about the mPFC.^9^^,^^49^ As a first preliminary analysis, we reconstructed hemoglobin response images; however, due to the brief event durations necessitated by this event-related design (2 s) and inherent noise in infant NIRS, group-level images were noisy. Despite this, this preliminary analysis (not accounting for engagement) revealed higher levels of activation in the frontal lobe for unexpected [Fig. 5(b)] compared with expected [Fig. 5(a)] conditions, with the strongest differences in activation appearing close to the midline [Fig. 5(c)]. As this simple analysis averaged across all trials for a given condition without considering individual trial-by-trial variability or task engagement, we chose not to statistically test the contrast at the image-level but present the images below for visualization purposes.

### 3.3 Region of Interest Analysis

**Fig. 6:**
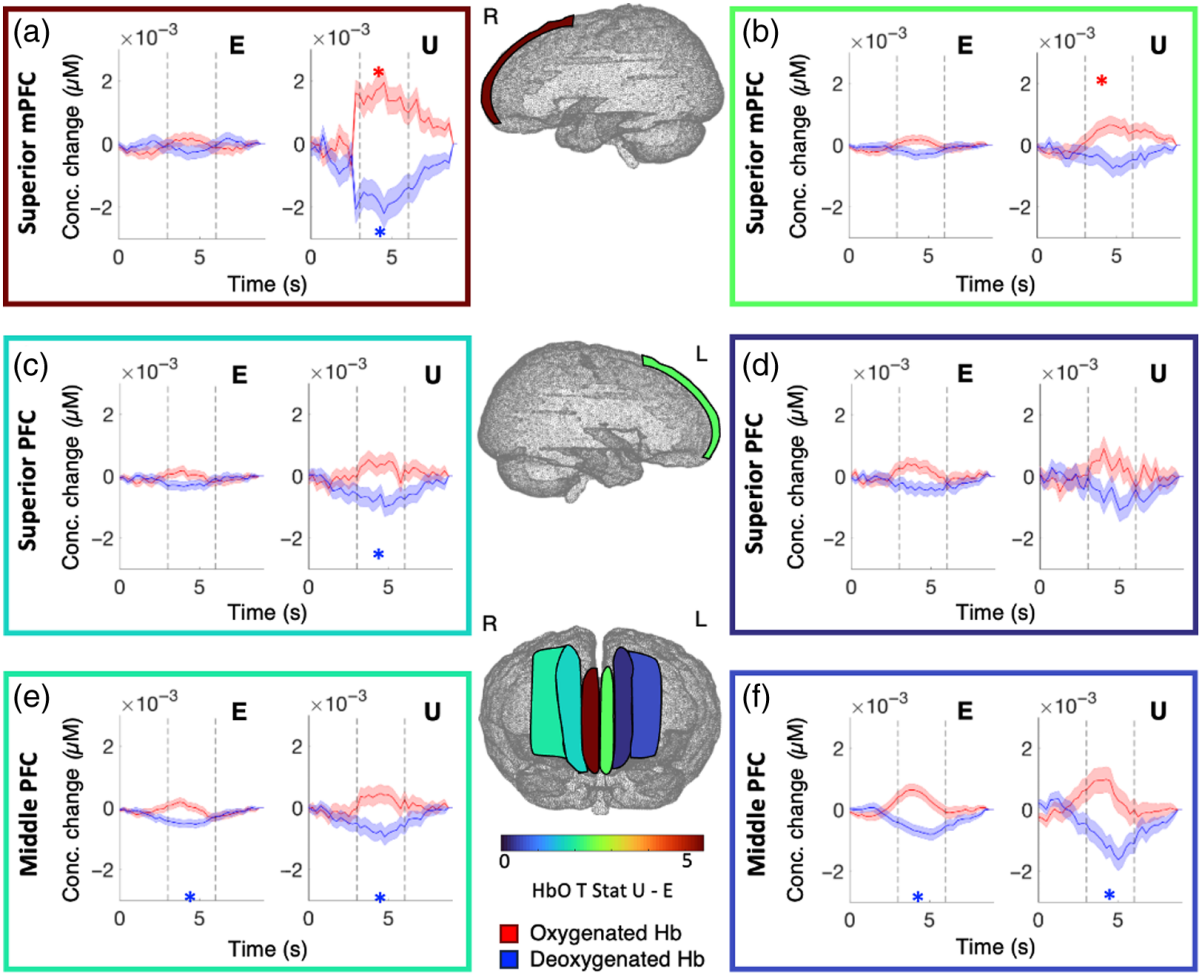
Results for the frontal ROI analysis projected on a 6-month cortical mesh. Hemodynamic responses to expected (E) and unexpected (U) stimuli are shown for oxyhemoglobin (HbO, red curves) and deoxygenated hemoglobin (HbR, blue curves) for the right medial prefrontal cortex (a), left medial prefrontal cortex (b), right prefrontal cortex (c), and left prefrontal cortex (d), right middle prefrontal cortex (e), and left middle prefrontal cortex (f). Asterisks indicate significant activation (p<0.05). This analysis includes measures for infant engagement.

To account for infant task engagement in our analyses and to understand the contributions of different frontal regions to prediction error processing, we conducted a region of interest analysis including DRT as a parametric modulator (Fig. 6). There were significant effects within the left mSFG for to unexpected stimuli [HbO, t(1680)=2.647, p=0.025; HbR, t(1680)=−1.934, p=0.06] [Fig. 6(b); Table S3 in the Supplementary Material]. There was significantly more activation for unexpected stimuli than expected stimuli in this region [HbO, t(1680)=2.678, p=0.02; HbR, t(1680)=−1.712] [Fig. 6(b), Table S4 in the Supplementary Material]. The right mPFC showed the same pattern of activity to unexpected stimuli as the left [HbO, t(1680)=5.541, p<0.000; HbR, t(1680)=−4.888, p<0.000], with no significant response to expected stimuli. The contrast of unexpected stimuli compared with expected showed significantly more activity for unexpected events [HbO, t(1680)=2.762, p=0.022; HbR, t(1680)=−2.835, p=0.028]. There was also significant HbR activation in the right mSFG [t(1680)=−2.459, p=0.021 [Fig. 5(a), Table S4 in the Supplementary Material]. There was a bilateral HbR response in the middle PFC for both expected [L t(1680)=−3.487, p=0.003; R t(1680)=−3.00, p=0.008] and unexpected [L t(1680)=−2.704, p=0.018; R t(1680)=−2.612, p=0.018] events [Figs. 6(e) and 6(f); Table S3 in the Supplementary Material]. When an equivalent ROI analysis was conducted that does not include infant engagement as an additional regressor, there were no significant differences in responses between expected and unexpected stimuli in the frontal lobe—see the Supplemental Material for full details.

### 3.4 Relationship to Maternal Trait Anxiety

**Fig. 7:**
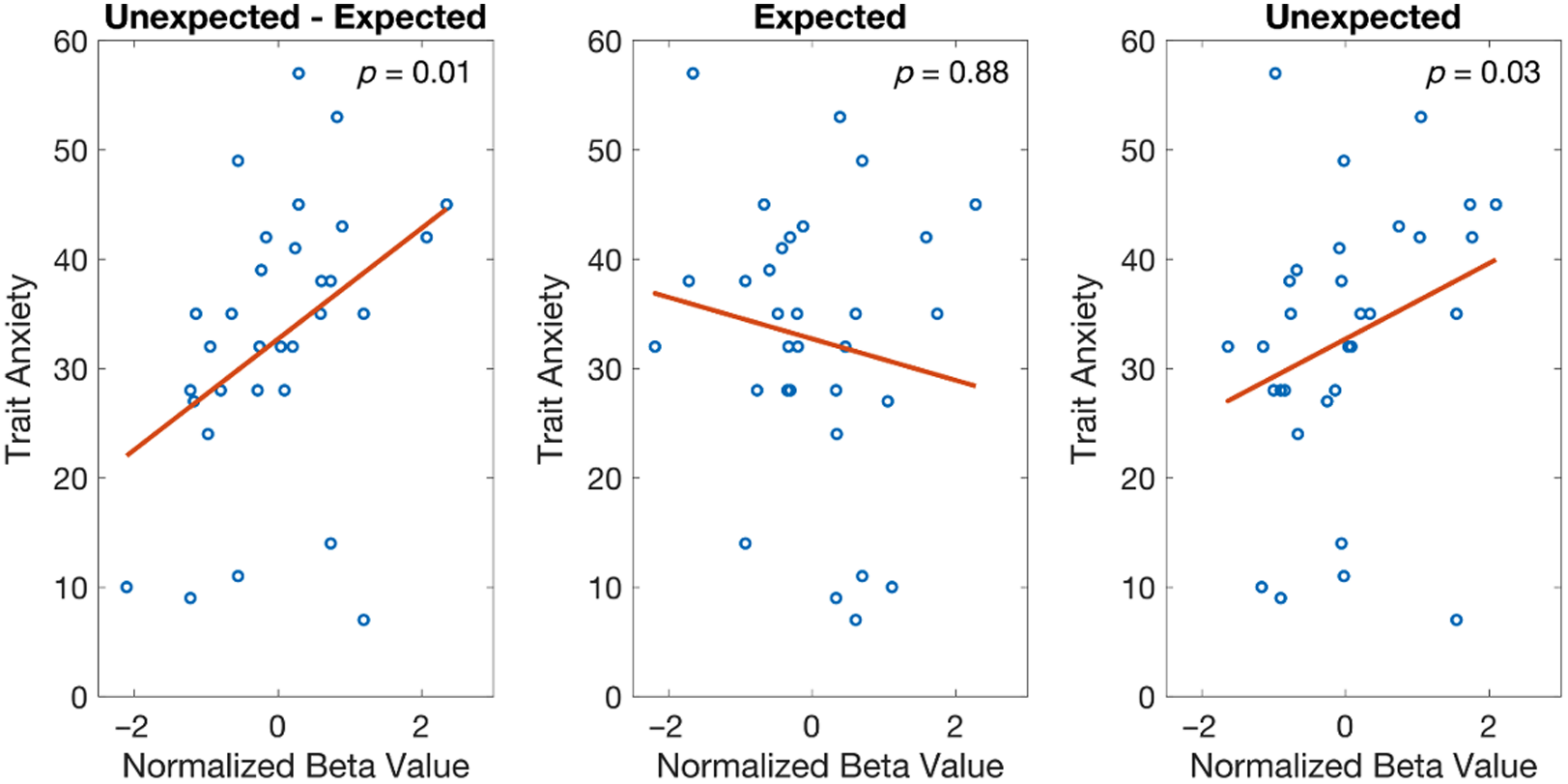
Relationship between infants’ normalized beta values in the right mPFC and their mothers’ trait anxiety levels as determined by the equation ′beta ∼−1 + cond + cond:Trait Anxiety + (1|subject). There was a significant response to the contrast of event types. This is driven by the relationship between maternal trait anxiety and an infant’s response to unexpected stimuli but not expected stimuli.

**Fig. 8:**
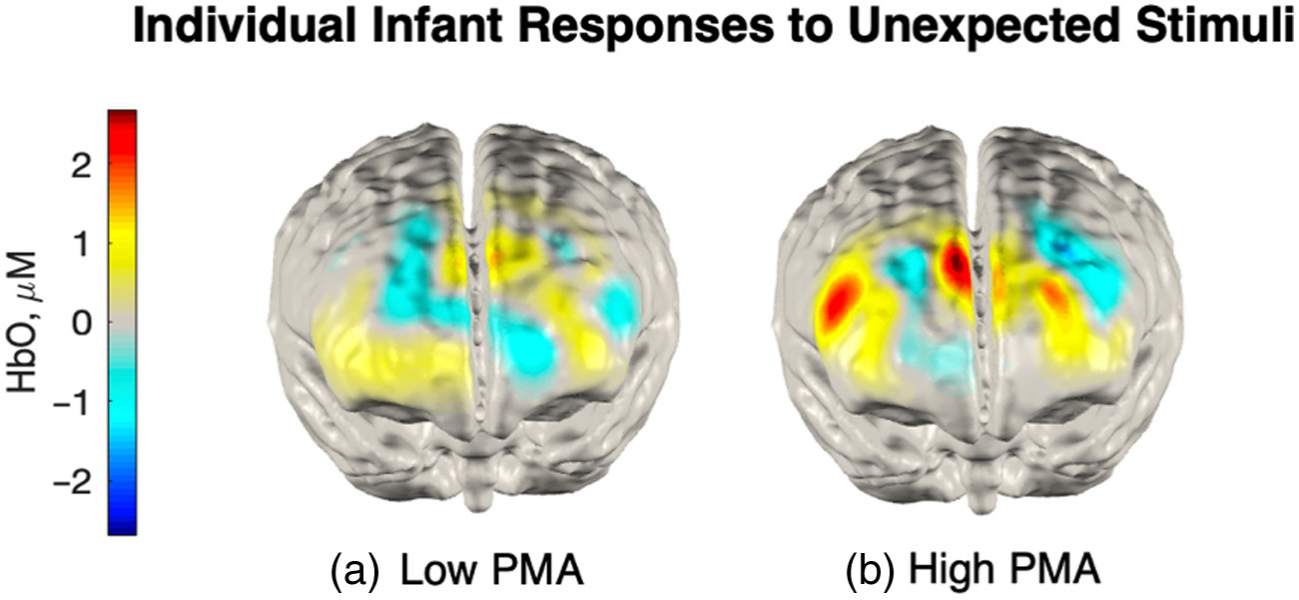
Example infant hemodynamic responses to unexpected stimuli for the infants whose mothers had the lowest and highest postnatal maternal anxiety scores in our sample (a) low postnatal maternal anxiety or (b) high postnatal maternal anxiety. The activation in the mPFC is seen for the infant whose mother had a high postnatal maternal anxiety score but not for the infant with a low postnatal maternal anxiety score. There is an additional response in the right dorsolateral PFC for the infant whose mother had high postnatal maternal anxiety, potentially indicating that this infant showed more selective attention.

Having established prediction error responses predominantly occur in the right infant mPFC, we next investigated whether these responses related to trait postnatal maternal anxiety levels (Fig. 7). We first examined HbO responses in the right mPFC to unexpected and expected events relative to baseline. There was a significant main effect of condition, with significantly more activation for unexpected compared with expected stimuli [HbO, t(1680)=2.762, p=0.022]. Post-hoc analyses revealed significant activation to unexpected stimuli [b=0.00124, SE=0.00051, t(46)=2.46, p=0.018] but not to expected stimuli [b=0.00026, SE=0.00038, t(46)=0.69, p=0.50]. This pattern indicates that the mPFC shows differential activation to unexpected vs expected events. Next, we assessed the role of trait anxiety in predicting cortical responses. A significant relationship was found between trait anxiety and HbO responses to unexpected stimuli in the right mPFC, b=0.00016, SE=0.00007, t(46)=2.20, p=0.033, indicating that higher levels of trait anxiety were associated with increased neural responses to unexpected events (Fig. 7). By contrast, state anxiety did not modulate cortical responses to unexpected events, b=6.16e−05, SE=7.40e−05, t(23)=0.83, p=0.41. For example, we show the frontal responses to unexpected stimuli for an infant whose mother has low postnatal maternal anxiety [Fig. 8(a)] and an infant whose mother has high postnatal maternal anxiety [Fig. 8(b)]. However, it would not be appropriate to statistically test if these differences in the mPFC between the two infants could predict maternal trait anxiety.

A linear regression was conducted to examine the relationship between DTR and maternal trait anxiety, considering the moderating effect of condition (expected versus unexpected). The results revealed that DTR was a significant predictor of trait anxiety, [b=1.74, SE=0.32, t(668)=−5.42, p<0.001], indicating that higher DTR was associated with lower trait anxiety scores. However, the main effect of condition (unexpected versus expected) was not significant, b=−0.48, SE=0.80, t(668)=−0.60, p=0.549, and there was no significant interaction between DTR and condition, b=−0.13, SE=0.86, t(668)=−0.15, p=0.883. These findings suggest that the relationship between DTR and trait anxiety did not differ between the expected and unexpected conditions (Fig. S5 in the Supplementary Material).

A logistic regression analysis examined whether infant mPFC responses could predict maternal clinical anxiety levels (STAI>40). There was no significant relationship for the contrast of unexpected–expected [T(23)=1.86; p=0.063], or for expected events compared with baseline [T(23)=0.874; p=0.38]. However, there was a significant relationship for unexpected events predicting clinically diagnosable postnatal maternal anxiety levels [T(23)=2.19; p=0.028].

### 3.5 Responses Beyond the Frontal Cortex

Although the primary aim of this study was to optimize the optode array for dense coverage of the frontal cortex, we did also have limited occipital coverage in an effort to recreate analyses of visual cortical responses seen in prior research (Emberson et al.^24^). However, the modifications made to the paradigm in service of an event-related design and gaze-tracking compromised the ability to cleanly measures visual responses. Our baseline was a video with moving, popping bubbles and our event durations were brief (2 s), meaning that our window of analysis was catching the response to the baseline. For visual cortical regions, this is not optimal and created what appears to be inverted responses (Fig. S1 in the Supplementary Material). In addition, the measurements of the visual cortices had an average power of 0.77 for the contrast of unexpected compared to expected events, whereas the regions in the superior frontal lobe had an average power of 0.98 (Tables S4 and S5 in the Supplementary Material).

## 4 Discussion and Conclusion

This study provides the first evidence that prediction error signals can be detected in the infant medial prefrontal cortex using high-density diffuse optical tomography and that these responses vary as a function of post-natal maternal anxiety. By combining an event-related design with concurrent eye-tracking, we were able to capture trial-by-trial variability in hemodynamic responses and prediction errors in 6- to 8-month-old infants, revealing that a greater response to unexpected events in the mPFC was associated with higher maternal trait anxiety. These findings extend predictive processing accounts of anxiety to early development and raises the intriguing possibility that alterations in infants’ neural encoding of surprise may be a candidate mechanism through which early environmental risk contributes to anxiety vulnerability.

Consistent with our hypotheses, we observed increased activation in the mPFC in response to unexpected, relative to expected, events. Furthermore, by capitalizing on the improved spatial resolution of HD-DOT, we were able to examine sub-regions of the prefrontal cortex with unprecedented anatomical specificity, localizing these prediction error responses to medial sources specifically. This aligns with prior work implicating the mPFC in expectation violation and volatility estimation in adults,^19^^,^^21^ and with developmental evidence in infancy showing that the mPFC emerges as a hub in the default mode network by 1 year of age.^50^ Our findings also build on prior work showing that by 6 to 8 months, infants are not only sensitive to violations of probabilistic regularities,^24^^,^^51^^,^^52^ but that—similar to adults—this processing recruits frontal cortical systems involved in learning and updating beliefs about the environment.

Notably, these mPFC responses were only detectable when we incorporated eye-tracking-derived measures of infant engagement into our models. Without controlling for attention, no significant differences were observed between expected and unexpected conditions (see Supplemental Material). Our finding of greater activation to unexpected events when controlling for dwell time actually provides evidence against a simple visual attention explanation. If the differences were purely due to visual stimulation, we would expect the opposite pattern (greater activation to expected trials with visual stimuli). This highlights the importance of accounting for fluctuations in infant attention when analyzing neuroimaging data, especially in tasks where stimulus salience or repetition effects may influence engagement over time. In fMRI research, the inclusion of trial-by-trial parametric modulators is standard practice, given that the BOLD signal is expected to vary as a function of cognitive state.^53^ In higher-order regions such as the frontal cortex, failing to model attention may simply reduce statistical power to detect true effects. However, in sensory regions, ignoring attention could lead to spurious results. For instance, if an infant shifts their attention away from the screen toward something novel in the environment, this may elicit stronger visual responses, similar to a prediction error, but unrelated to the task. If “attention grabbers” (e.g., bubbles, rattles, puppets) are used to regain attention when nothing is on the screen (e.g., during stimulus omissions), this could also create spurious prediction error responses. Recent studies have attempted to address these confounds with gaze-contingent paradigms that ensure infant engagement (without the need for experimenter interaction) and prediction errors elicited by the nature of the unexpected stimulus rather than its omission.^54^

Developmental studies have traditionally relied on manual video coding to assess infant attention.^55^ However, this method is unnecessarily labor-intensive and prone to error.^56^ Critically, video coding cannot easily detect covert shifts in attention—so a trial may be incorrectly marked as valid in the absence of clear overt signs of movement. By contrast, eye-tracking provides an objective and precise way to measure infant gaze, allowing researchers to capture engagement moment-to-moment and control for its effects in analyses. This approach is especially valuable when trial-by-trial attention fluctuations are theoretically important. This has become recommended practice in EEG studies,^57^ and we argue that similar integration with eye-tracking should be adopted in optical imaging studies, where objective measures of engagement remain underutilized. Eye-tracking also facilitates novel task designs in infancy research, including looking-dependent paradigms that require overt responses and trigger trials only when infants are attending.^58^ These designs can increase experimental efficiency by reducing the number of unusable trials and enhancing data quality. Moreover, by ensuring neural responses are time-locked to or modulated by meaningful behavioral engagement, such approaches enable more precise interpretations of neural signals. As optical imaging methods such as HD-DOT and fNIRS continue to expand in developmental research, this opens new avenues for investigating the dynamic interplay between attention and brain activity in early life.

A central finding of this study is that infants of mothers with higher trait anxiety exhibited stronger mPFC responses to unexpected events. This association was specific to trait (rather than state) anxiety. Furthermore, an infant’s right mPFC response to surprise predicted whether their mother met the clinical threshold for anxiety on the STAI. These findings suggest that infants raised in high-anxiety environments may develop heightened neural sensitivity to surprise or uncertainty, potentially reflecting early adaptations to an unpredictable or threat-sensitive caregiving context. This is consistent with theoretical models positing that early exposure to unpredictable environments may bias infants toward hyper-responsivity to environmental change.^59^ It also parallels work in older children and adults linking anxiety to altered prediction error signaling in the mPFC and related fronto-subcortical circuits.^21^^,^^60^ Although the current study cannot establish causality or directionality, the association between postnatal maternal anxiety and infant mPFC responses raises the possibility that alterations in prediction error processing may serve as an early neurocognitive marker of anxiety risk.

Methodologically, this study advances the field by demonstrating the feasibility of using HD-DOT to image the infant frontal cortex with sufficient spatial resolution to detect functionally specific responses. Our optode array allowed for dense sampling of frontal regions and, combined with an event-related design and concurrent eye-tracking, enabled precise analysis of trial-level neural responses in awake infants. Importantly, the use of a GLM analysis approach that included infant engagement provides a framework for future infant neuroimaging studies aiming to disentangle cognitive processes from attentional confounds. Nevertheless, several limitations should be noted. First, the final sample size for this dataset was modest (N=31), and the subsample with maternal anxiety data even smaller (n=25). Although these sample sizes are standard for infant fNIRS research^61^ and likely sufficient to detect medium-large effects, larger samples are recommended to examine brain–behavior correlations.^62^ Replication in larger and more diverse cohorts is needed to validate and extend these findings. Second, our measure of maternal anxiety was based on self-report and did not include diagnostic interviews or contextual information about caregiving behavior, which may also shape infant brain development. Future studies should aim to replicate these findings alongside observational or ecological measures of maternal affective behavior and environmental predictability. In addition, we did not assess prenatal maternal stress or anxiety, which limits our ability to determine whether the observed effects are specifically related to postnatal maternal anxiety or might reflect continuity from prenatal psychological states. Future studies should include both prenatal and postnatal measures to better understand the temporal specificity of these associations.

Our primary focus was on frontal prediction error responses, and the study was not optimally designed to test sensory regions owing to the audio-visual baseline and the short event durations in this task design. In addition, statistical power was lower in the occipital array relative to the frontal array (Tables S4 and S5 in the Supplementary Material), most likely due to differences in hair density between the front and back of the head (sensitivity profile shown in Fig. S5 in the Supplementary Material). In addition, our array was vertically arranged, capturing only the primary visual cortex and parts of the parietal cortex, whereas prior research using lower-resolution systems extended to ventral/secondary visual cortices and into the temporal lobe.^24^ As such, our conclusions are restricted to the frontal cortex, though future work with broader array coverage and task designs optimized for sensory contrasts might be able to interrogate the dynamics between sensory and frontal regions during learning.

Our findings suggest that altered prediction error processing in the mPFC may emerge in infancy as a function of maternal anxiety and could represent a target mechanism for early intervention. If replicated, mPFC responses to unexpected events have the potential to be useful as a neurobiological marker of heightened sensitivity to environmental unpredictability—an endophenotype of anxiety risk. However, longitudinal studies are now needed to examine whether early mPFC prediction error signals predict later anxiety symptoms, cognitive flexibility, or emotional regulation. Finally, our work contributes to the growing body of research suggesting that predictive processing frameworks may provide a unifying account of cognitive-affective development across typical and atypical trajectories.^51^ Future research should examine how prediction error-related signals in infancy contribute to the maturation of higher-order functions such as expectation formation, uncertainty resolution, and self-regulation. Such work will be essential for building mechanistic accounts of how the brain constructs and updates models of the world across early development, and how these processes scaffold emerging cognitive-affective capacities.

In summary, by leveraging a multimodal approach that integrates gaze-tracking alongside advanced DOT methodologies, we unveil novel insights into infant prediction and learning. Moreover, our findings underscore the pivotal role of postnatal maternal mental health in shaping early brain responses to unpredictability, underscoring the need for comprehensive support systems to promote maternal well-being and, by extension, infant neurodevelopment.

## Supplementary Material

10.1117/1.NPh.12.3.035013.s01

## Data Availability

All analysis was completed using a combination of publicly available toolboxes, as described in the paper above. Gaze-tracking and HD-DOT data can be found on Dr Billing’s GitHub (https://www.github.com/adnb113).
