# Supplementary material for: Maternal anxiety shapes prediction error responses in the infant brain
Source: Neurophotonics. 2025 Sep 16;12(3):035013. doi: 10.1117/1.NPh.12.3.035013 (PMC12440255; doi:10.1117/1.NPh.12.3.035013)
Supplement: Supplementary file 1 [file NPh_012_035013_SD001.docx]

**Supplementary figures and tables**

**Supplemental Figure 1:** Q–Q plot and histogram of residuals from the Condition model. While the Shapiro–Wilk test was significant, the residuals showed a reasonably linear distribution with minor tail deviations, supporting the validity of the linear mixed model approach.

**Supplementary Figure 2:** Q–Q plot and histogram of residuals from the habituation model. Residuals again showed minor deviations from normality, justifying the model’s use despite bounded response data.


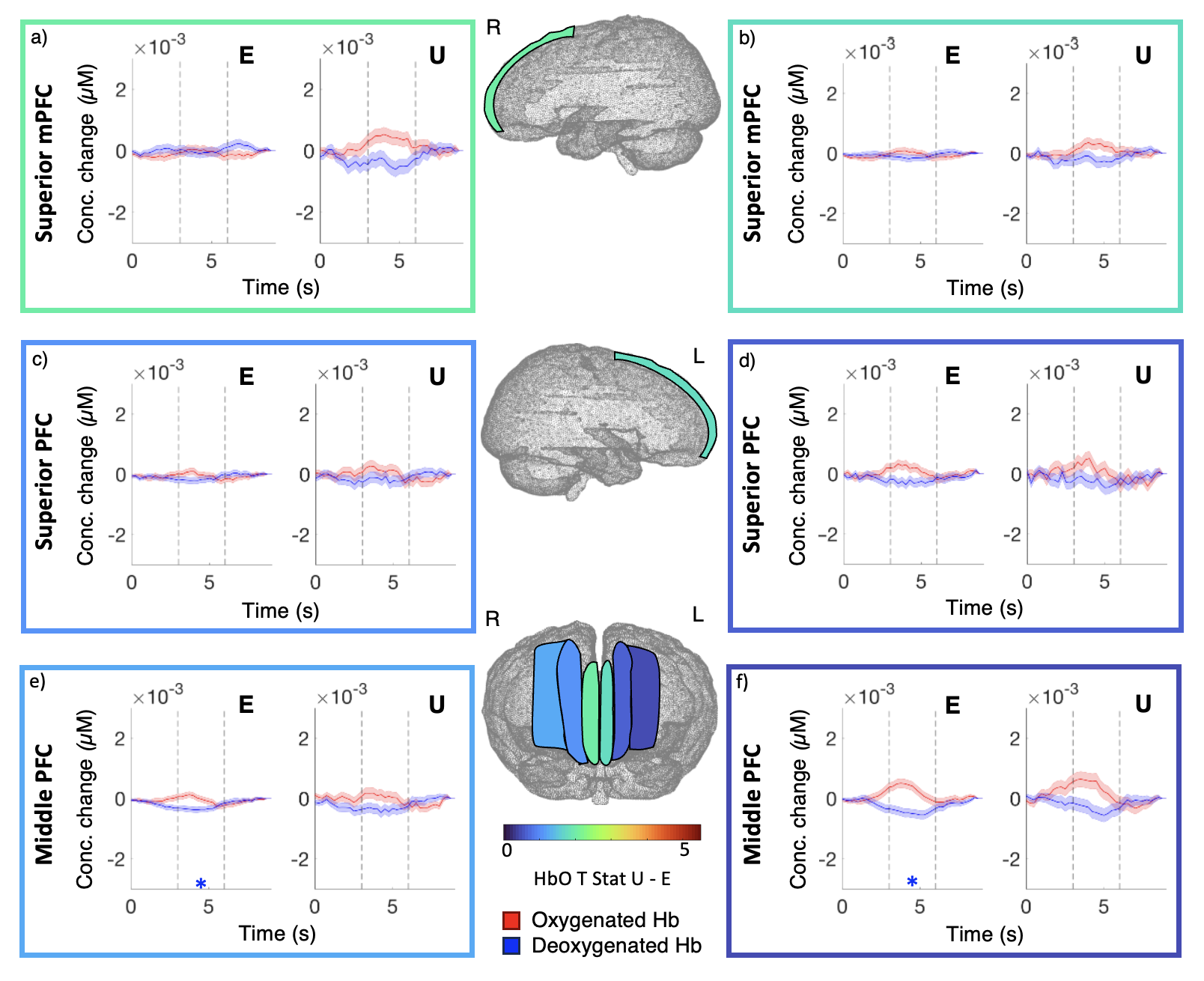


**Supplementary Figure 3: Results for the frontal ROI analysis projected on a 6-month cortical mesh.** Hemodynamic responses to expected and unexpected stimuli are shown for the right medial prefrontal cortex (a), left medial prefrontal cortex (b), right prefrontal cortex (c), and left prefrontal cortex (d), right middle prefrontal cortex (e) and left middle prefrontal cortex (f). This analysis does not include measures for infant engagement, and there are no significantly different responses between expected and unexpected conditions.

**
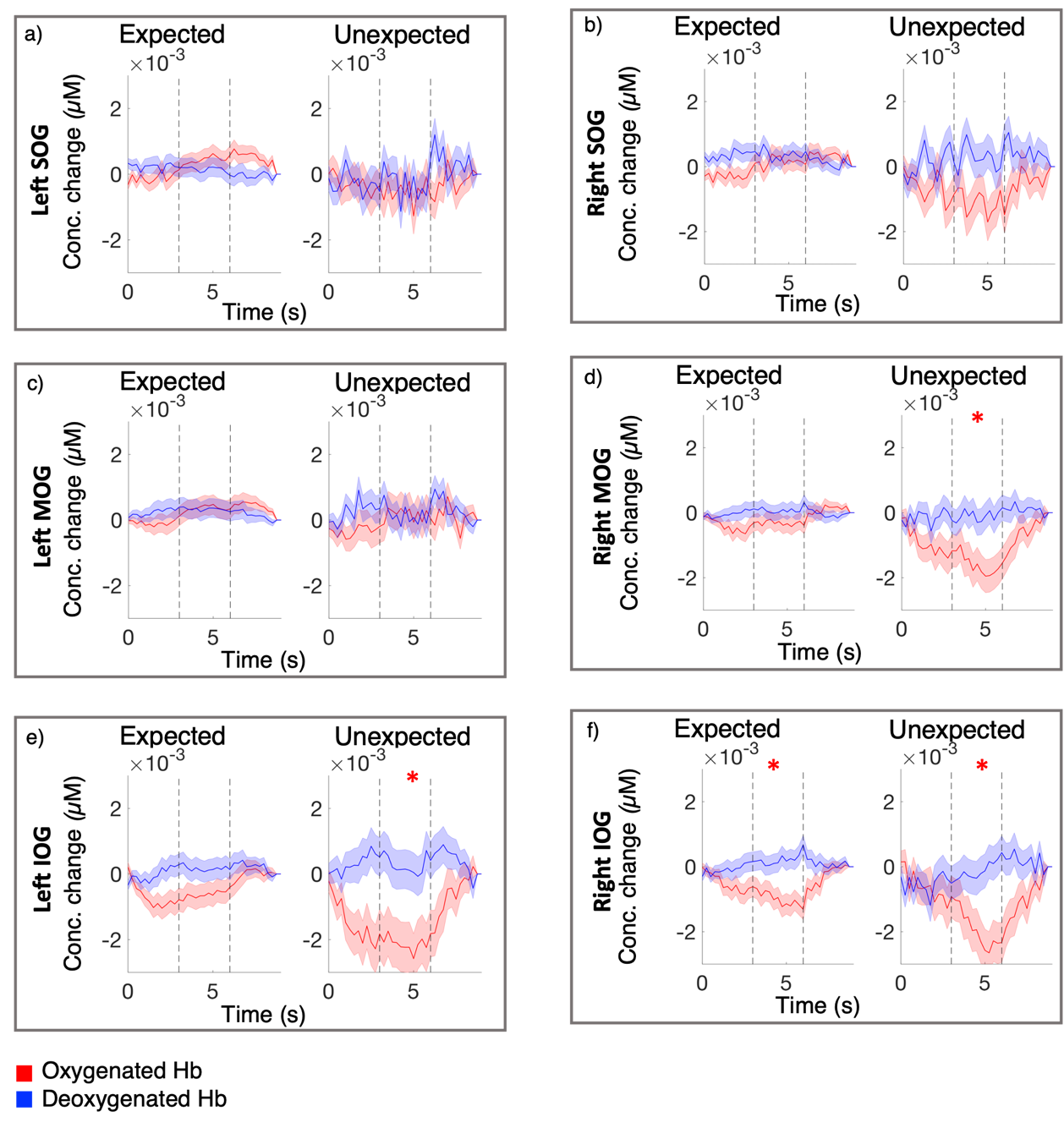
**

**Supplementary Figure 4:** Responses occipital lobe when weighted by gaze.


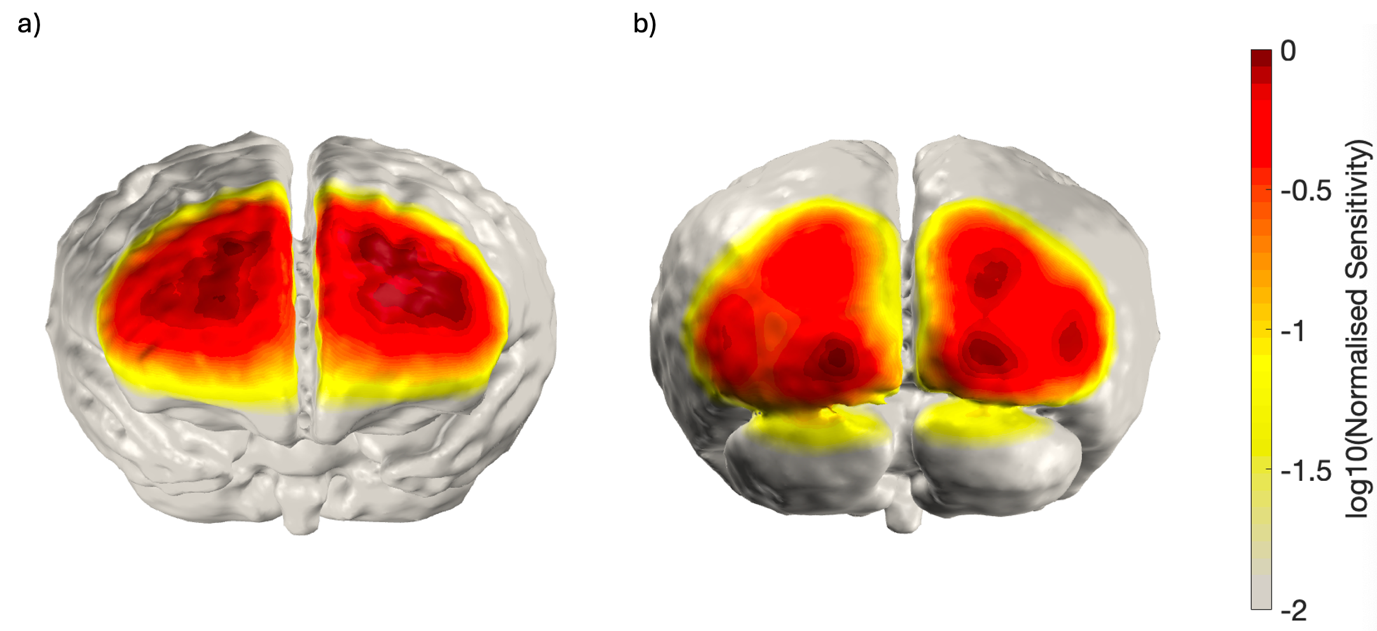


**Supplementary Figure 5:** Relative sensitivity of the frontal and occipital lobes for a given randomly selected individual (Figure 1).

Supplementary Table 1: Frontal lobe. Expected and unexpected activation from 3 to 6 seconds when compared to baseline with no gaze-tracking included in the analysis.

| **Gaze-tracking not included** | T-Stat | Corrected p | Power |
| --- | --- | --- | --- |
| **Expected : HbO** |  |  |  |
| 'frontal_sup_l' | 0.913 | 0.504 | 0.971 |
| 'frontal_sup_r' | -1.036 | 0.504 | 0.979 |
| 'frontal_sup_medial_l' | 0.059 | 0.990 | 1.000 |
| 'frontal_sup_medial_r' | 0.028 | 0.990 | 1.000 |
| 'frontal_mid_l' | 1.699 | 0.504 | 0.939 |
| 'frontal_mid_r' | -1.135 | 0.504 | 0.945 |
| **Expected : HbR** |  |  |  |
| 'frontal_sup_l' | -1.054 | 0.615 | 1.000 |
| 'frontal_sup_r' | -0.228 | 0.810 | 1.000 |
| 'frontal_sup_medial_l' | -0.560 | 0.729 | 0.974 |
| 'frontal_sup_medial_r' | 0.831 | 0.679 | 0.967 |
| 'frontal_mid_l' | -2.747 | 0.030 | 1.000 |
| 'frontal_mid_r' | -2.584 | 0.030 | 1.000 |
| **Unexpected : HbO** |  |  |  |
| 'frontal_sup_l' | 1.356 | 0.263 | 0.948 |
| 'frontal_sup_r' | 0.558 | 0.570 | 0.989 |
| 'frontal_sup_medial_l' | 1.736 | 0.196 | 1.000 |
| 'frontal_sup_medial_r' | 2.020 | 0.196 | 1.000 |
| 'frontal_mid_l' | 1.751 | 0.196 | 0.887 |
| 'frontal_mid_r' | 0.567 | 0.570 | 0.927 |
| **Unexpected : HbR** |  |  |  |
| 'frontal_sup_l' | -1.298 | 0.271 | 1.000 |
| 'frontal_sup_r' | -0.433 | 0.628 | 1.000 |
| 'frontal_sup_medial_l' | -1.199 | 0.271 | 0.960 |
| 'frontal_sup_medial_r' | -1.758 | 0.271 | 0.953 |
| 'frontal_mid_l' | -1.273 | 0.271 | 1.000 |
| 'frontal_mid_r' | -1.672 | 0.271 | 1.000 |

Supplementary Table 2: Frontal lobe. The contrast between activation associated with unexpected and expected events for a 3 to 6 second block with no gaze-tracking included in the analysis.

|  | T-Stat | Corrected p | Power |
| --- | --- | --- | --- |
| **Unexpected - Expected : HbO** |  |  |  |
| 'frontal_sup_l' | 0.774 | 0.467 | 0.930 |
| 'frontal_sup_r' | 1.206 | 0.370 | 0.992 |
| 'frontal_sup_medial_l' | 1.715 | 0.259 | 1.000 |
| 'frontal_sup_medial_r' | 2.018 | 0.259 | 1.000 |
| frontal_mid_l' | 0.727 | 0.467 | 0.856 |
| frontal_mid_r' | 1.159 | 0.370 | 0.918 |
| **Unexpected - Expected : HbR** |  |  |  |
| 'frontal_sup_l' | -0.712 | 0.850 | 1.000 |
| 'frontal_sup_r' | -0.340 | 0.850 | 1.000 |
| 'frontal_sup_medial_l' | -0.917 | 0.850 | 0.951 |
| 'frontal_sup_medial_r' | -2.244 | 0.150 | 0.938 |
| frontal_mid_l' | 0.516 | 0.850 | 1.000 |
| frontal_mid_r' | -0.190 | 0.850 | 1.000 |

Supplementary Table 3: Frontal lobe. Expected and unexpected activation from 3 to 6 seconds when compared to baseline with gaze-tracking included in the analysis.

| **Gaze-tracking included** | T-Stat | Corrected p | Power |
| --- | --- | --- | --- |
| **Expected : HbO** |  |  |  |
| 'frontal_sup_l' | 1.270 | 0.613 | 0.962 |
| 'frontal_sup_r' | -0.431 | 0.667 | 0.983 |
| 'frontal_sup_medial_l' | 0.679 | 0.667 | 1.000 |
| 'frontal_sup_medial_r' | 0.509 | 0.667 | 1.000 |
| 'frontal_mid_l' | 1.939 | 0.316 | 0.923 |
| 'frontal_mid_r' | -0.696 | 0.667 | 0.944 |
| **Expected : HbR** |  |  |  |
| 'frontal_sup_l' | -1.753 | 0.159 | 1.000 |
| 'frontal_sup_r' | -0.973 | 0.397 | 1.000 |
| 'frontal_sup_medial_l' | -1.227 | 0.330 | 0.967 |
| 'frontal_sup_medial_r' | -0.114 | 0.910 | 0.954 |
| 'frontal_mid_l' | -3.487 | 0.003 | 1.000 |
| 'frontal_mid_r' | -3.000 | 0.008 | 1.000 |
| **Unexpected : HbO** |  |  |  |
| 'frontal_sup_l' | 0.919 | 0.358 | 0.928 |
| 'frontal_sup_r' | 1.562 | 0.178 | 1.000 |
| 'frontal_sup_medial_l' | 2.647 | 0.025 | 1.000 |
| 'frontal_sup_medial_r' | 5.541 | 0.000 | 1.000 |
| 'frontal_mid_l' | 1.429 | 0.184 | 0.881 |
| 'frontal_mid_r' | 1.699 | 0.178 | 0.929 |
| **Unexpected : HbR** |  |  |  |
| 'frontal_sup_l' | -1.628 | 0.104 | 1.000 |
| 'frontal_sup_r' | -2.459 | 0.021 | 0.949 |
| 'frontal_sup_medial_l' | -1.934 | 0.064 | 0.909 |
| 'frontal_sup_medial_r' | -4.888 | 0.000 | 0.908 |
| 'frontal_mid_l' | -2.704 | 0.018 | 1.000 |
| 'frontal_mid_r' | -2.612 | 0.018 | 1.000 |

Supplementary Table 4: Frontal lobe. The contrast between activation associated with unexpected and expected events for a 3 to 6 second block with gaze-tracking included in the analysis.

|  | T-Stat | Corrected p | Power |
| --- | --- | --- | --- |
| **Unexpected - Expected : HbO** |  |  |  |
| 'frontal_sup_l' | 0.218 | 0.827 | 0.916 |
| 'frontal_sup_r' | 1.668 | 0.143 | 1.000 |
| 'frontal_sup_medial_l' | 2.678 | 0.022 | 1.000 |
| 'frontal_sup_medial_r' | 2.762 | 0.022 | 1.000 |
| 'frontal_mid_l' | 0.470 | 0.561 | 0.862 |
| 'frontal_mid_r' | 2.014 | 0.088 | 0.921 |
| **Unexpected - Expected : HbR** |  |  |  |
| 'frontal_sup_l' | -0.785 | 0.519 | 1.000 |
| 'frontal_sup_r' | -2.027 | 0.090 | 0.947 |
| 'frontal_sup_medial_l' | -1.712 | 0.131 | 0.896 |
| 'frontal_sup_medial_r' | -2.835 | 0.028 | 0.903 |
| 'frontal_mid_l' | 0.517 | 0.605 | 0.890 |
| 'frontal_mid_r' | -0.014 | 0.090 | 0.921 |

Supplementary Table 5: Occipital lobe. Expected and unexpected activation from 3 to 6 seconds when compared to baseline with gaze-tracking included in the analysis.

|  | T-Stat | Corrected p | Power |
| --- | --- | --- | --- |
| **Expected : HbO** |  |  |  |
| 'occipital_inf_l' | -1.574 | 0.174 | 0.916 |
| 'occipital_inf_r' | -3.303 | 0.006 | 0.930 |
| 'occipital_mid_l' | 1.576 | 0.174 | 0.972 |
| 'occipital_mid_r' | -0.694 | 0.488 | 0.915 |
| 'occipital_sup_l' | 2.278 | 0.069 | 1.000 |
| 'occipital_sup_r' | 1.453 | 0.176 | 0.954 |
| **Expected : HbR** |  |  |  |
| 'occipital_inf_l' | 0.308 | 0.758 | 1.000 |
| 'occipital_inf_r' | 0.868 | 0.385 | 1.000 |
| 'occipital_mid_l' | 0.583 | 0.560 | 1.000 |
| 'occipital_mid_r' | 0.483 | 0.629 | 1.000 |
| 'occipital_sup_l' | -0.088 | 0.930 | 0.991 |
| 'occipital_sup_r' | 1.314 | 0.189 | 1.000 |
| **Unexpected : HbO** |  |  |  |
| 'occipital_inf_l' | -2.683 | 0.015 | 0.791 |
| 'occipital_inf_r' | -3.046 | 0.014 | 0.809 |
| 'occipital_mid_l' | 0.116 | 0.908 | 0.834 |
| 'occipital_mid_r' | -2.827 | 0.014 | 0.807 |
| 'occipital_sup_l' | -0.677 | 0.598 | 0.961 |
| 'occipital_sup_r' | -1.412 | 0.237 | 0.914 |
| **Unexpected : HbR** |  |  |  |
| 'occipital_inf_l' | 0.035 | 0.972 | 1.000 |
| 'occipital_inf_r' | -0.967 | 0.334 | 1.000 |
| 'occipital_mid_l' | 0.295 | 0.768 | 1.000 |
| 'occipital_mid_r' | -0.892 | 0.373 | 1.000 |
| 'occipital_sup_l' | 0.307 | 0.759 | 1.000 |
| 'occipital_sup_r' | 0.802 | 0.423 | 1.000 |

**Supplementary Figure 6: The relationship between an individual’s average DTR per condition and their mother’s trait anxiety scores.** As DTR is simply looking at the time spend looking at the screen there was no correlation with maternal behaviour for either condition.
